# Review: Practical Use of n-3 Fatty Acids to Improve Reproduction Parameters in the Context of Modern Sow Nutrition

**DOI:** 10.3390/ani10071141

**Published:** 2020-07-06

**Authors:** Róbert Roszkos, Tamás Tóth, Miklós Mézes

**Affiliations:** 1 Department of Nutrition, Szent István University, 2103 Gödöllő, Hungary; mezes.miklos@mkk.szie.hu; 2 ADEXGO Ltd., 8230 Balatonfüred, Hungary; tamas.toth@adexgo.hu

**Keywords:** swine, nutrition, n-3 fatty acids, reproduction

## Abstract

**Simple Summary:**

The use of n-3 fatty acids could have many favourable aspects considering the nutrition of mammals, as can be seen from studies carried out on humans or livestock animals. Concerning large-scale pig farms, the reproduction performance could be made more balanced at a high level by enhancing efficacy and decreasing the ecological footprint of pork production. In this review, we attempt to identify specific periods in the sow production cycle in which the feeding of n-3 fatty acids returns an investment, in addition to demonstrating the importance of the dosage and proportion of n-6 and n-3 fatty acids, originating from different nutritional sources.

**Abstract:**

The effects of long-chain polyunsaturated fatty acids (LC PUFAs) have been frequently investigated in sows because the profitability of pig production depends mainly on reproduction performance. In feeding trials, different sources and doses of n-3 PUFAs-rich feeds were used with various breeds and stages of production; however, a discrepancy in the response of n-3 PUFAs on sow reproduction has been observed. According to the results of the previous studies, n-3 fatty acids can postpone the time of parturition, decreasing the synthesis of prostaglandins, which are necessary for uterus contraction during labour. These effects could also be useful during the post-weaning period when low prostaglandin levels are indispensable for embryo survival. The n-3 fatty acids fed during the lactation period secreted in milk, may improve piglet performance. In this review, we will focus on the contradictory results of previous studies concerning practical swine nutrition. The main purpose of the review is to highlight those periods of swine breeding when the use of n-3 fatty acids may be advantageous in case of the deficiency of these essential nutrients. In finding the appropriate dose of n-3 PUFAs in terms of sow nutrition, the n-6 PUFAs levels in the given feeds must be taken into account to ensure that there are no significant reductions in the final n-6/n-3 ratio. Despite the numerous previous field trials, there are no current feeding recommendations available for PUFAs in swine nutrition. Hence, more research is required in different practical feeding situations to certify the assumptions and conclusions of this review.

## 1. Introduction

In recent years, large-scale swine production has gone through a process of transformation. Modern hyperprolific breeds have started to spread all around the world. Besides large litter sizes, the resulting pigs have higher growth and fattening capability. The increased litter size, due to genetic selection for prolificacy, has coincided with an increase in stillborn rates and piglets born with lower viability because of lower individual birth weight, resulting in high pre-weaning mortality [1]. For example, the number of live piglets of the DanBred Landrace and DanBred Yorkshire breeds on day five reached 13.4 in 2019, and this number has been continuously increasing. Pre-weaning mortality until day five was 15% of the total piglets born (DanBred Breeding Program, 2020).

The high pre-weaning mortality rate is mainly attributable to the large litter size, which leads to the low individual weights of new-born piglets, who are also less developed, and low-vitality animals that are more likely to die during the first period of lactation. The first five days after birth are critical; most of the losses take place during this period.

Nutrition is one of the most crucial factors in large-scale swine production. According to Pennsylvania State University [2], a farrow-to-finish farm will spend 75% of its total expenses on feed compared to 50% for a farrow-to-feeder, and 65% for a feeder-to-finish farm. Satisfying the energy and nutrient requirements of hyperprolific sows significantly influences the effectiveness of (re)production and the longevity of sows. As reproduction biology is one of the most critical strengths of these “high-performance” modern breeds, supporting this through appropriate feeding is one of the main goals from an economic point of view. Numerous studies have demonstrated that adequate energy supplementation improves reproductive performance [3,4]. Due to the energy surplus, the secretion of follicle-stimulating hormone (FSH) and luteinizing hormone (LH) is enhanced, which has a beneficial effect on follicular growth and significantly influences the progesterone synthesis of corpus luteum (CL), following ovulation. 

The conventional method used to increase the energy concentration of the diet is through supplementation with fat or oil, as their energy concentration is 2–2.5 times higher than that of other nutrients. Moreover, the use of specialized fat sources that can be beneficial not only because of their high energy content, but also because of their positive effects on reproductive and other physiological processes [5]. The n-3 fatty acids belonging to the group of long-chain polyunsaturated fatty acids (LC PUFAs) have been studied for many years regarding their effects on swine production. In this review, the practical application of n-3 fatty acids is discussed, with a focus on the phases of the reproductive cycle to which they could contribute the most. The main purpose of the review is to highlight those periods of swine breeding when the use of n-3 fatty acids may be advantageous in case of deficiency of these essential nutrients.

## 2. Long-Chain Polyunsaturated Fatty Acids

### 2.1. Classification

LC PUFAs have been a focus of scientific research and practical nutrition for a long time. LC PUFAs are composed of at least 18 carbon atoms and contain at least two unsaturated double bonds in their acyl chain. PUFAs are classified according to the position of the first double bond, relative to the terminal methyl group. Consequently, the short names given to PUFAs show how far the first double bond is from the end of the molecule (e.g., the third carbon atom in the case of n-3 and sixth in the case of n-6 PUFAs) [6]. 

Among the various LC PUFAs, linoleic acid (LA; C18:2, n-6) and α-linolenic acid (ALA; C18:3, n-3) are considered essential for vertebrates because they are unable to synthesize them and must be acquired from their diet [7].

From the physiological point of view, as discussed later, the most important PUFAs are arachidonic acid (ARA; C20: 4, n-6), formed from LA, and eicosapentaenoic acid (EPA; C20: 5, n-3) and docosahexaenoic acid (DHA; C22: 6, n-3), both of which are formed from ALA.

### 2.2. Biological Functions and Transformation to Longer-Chain Fatty Acids

LC PUFAs have diverse effects in the body. They are involved in building the outer membranes of cells (phospholipid bilayer), preserving cell integrity and the fluidity of membranes supporting some intracellular signal transduction mechanisms. They are involved in the biosynthesis of certain hormones (e.g., prostanoids) and immune processes [8]. Moreover, they can affect the expression of specific genes that play a role in the regulation of lipid metabolism and cholesterol synthesis [9].

LC PUFAs can act on immune functions via ARA and EPA, which play key roles in the formation of eicosanoids, which, in turn, are essential chemical messengers. ARA is a precursor of the 2-series of prostaglandins (PGs), 2-series of thromboxanes (TXs), and 4-series of leukotrienes (LTs), whereas EPA leads to the formation of the 3-series of PGs, 3-series of TXs and 5-series of LTs [10]. These eicosanoids can exert pro- and anti-inflammatory effects, depending on the balance between their precursors, and also play key roles in the reproduction process, as discussed later. LA and ALA can be converted to longer-chained fatty acids by specific enzymes (such as microsomal desaturases and elongases). The elongation of the two types of LC PUFAs (n-6 and n-3) is carried out in parallel since the same enzymes are required for both, though the two pathways are not permeable [11]. As the metabolism of these two types of fatty acid is distinct, the quantity of each in the body can be directly influenced by their rate of inclusion in feeds.

The conversion rates of longer-chain PUFAs from their precursors (LA, ALA) are determined by the amounts of enzyme available for conversion, especially Δ5- and Δ6-desaturases. Shahidi and Ambigaipalan [12] found a 4% rate of conversion of ALA to longer-chain n-3 PUFAs, indicating the importance of ALA in sow feed. 

The sex-based differences in LC PUFAs metabolism are well known. In females, the conversion rate of ALA to EPA was shown to be 2.5-fold higher than in males, and it reaches about 20% [13]. The possible reason is that ALA is not used for energy production by beta-oxidation and remains available for conversion to EPA [14]. Another possible explanation could be the direct effect of oestrogen on conversion. Oestrogen causes higher DHA concentrations in females, probably by upregulating DHA synthesis from its precursors, mainly EPA [15]. The rate of conversion of ALA to DHA in females is thought to be as high as 9%, especially during pregnancy. It has been hypothesized that demands for DHA by the foetus may stimulate the metabolism of females to synthesize more of this fatty acid. The explanation of the conversion rate of ALA to other longer-chain n-3 fatty acids, such as EPA and DHA in females, could be their vital role in cell membrane functions and thus in the development of the foetal brain and cardiovascular and immune system [13,16].

There are comprehensive works and reviews in the literature, which introduce the chemistry, physiology and metabolism of LC PUFAs in more detail, also including the biological roles and backgrounds [7,12,17]. Additional information can be acquired from the work of Whathes et al. [3], who summarised the roles of LC PUFAs in female and male reproduction.

## 3. The Biological Function of LC PUFAs in Reproduction

According to Wathes et al. [3], LC PUFAs influence the reproductive performance in such a way that they positively affect follicle development and the expression of genes encoding the enzymes necessary for the formation of prostaglandins and sex steroids. The pig oocyte has high levels of LC PUFA, particularly the n-6 fatty acids, such as LA and ARA, indicating a local role in actively producing PGs [18].

LC PUFAs can pass through the placenta into the foetus and can be excreted through sow milk [19]. Due to a selective transportation process through the placenta, mainly from the second trimester onwards, a much higher concentration of ARA and DHA can be found in the foetus than in maternal blood in humans and swine [20].

Some positive effects of LC PUFAs on reproductive biology are linked to n-3 fatty acids (ALA, EPA, and DHA). These LC PUFAs can be incorporated into oocytes and increase the number and quality of follicles on the ovaries and are able to alter the expression of genes involved in prostaglandin biosynthesis, as observed in cattle and sheep [21,22].

Human studies have found that a higher maternal n-3/n-6 PUFA ratio is associated with a higher foetal growth rate and birth weight, possibly due to the longer duration of pregnancy [23]. Li et al. [24] reviewed the observations of 22 human studies. They revealed that the supplementation of the maternal diet with n-3 LC PUFA during pregnancy and/or lactation was generally associated with a significantly higher infant birth weight compared to the unsupplemented controls. Subgroup analysis showed that there was a higher birth weight in the group given a high dose of DHA (≥800 mg/day); hence, EPA treatment had a significant effect on birth weight, even at low doses.

LC PUFAs contribute to the improved recognition of pregnancy by the dam, such that early embryo death may be reduced. In ruminants, during the growth of embryos, a specific signal called interferon-τ (IFN-τ) is used to inform the maternal body about their existence, and this sign ensures the maintenance of pregnancy by suppressing prostaglandin F2α (PGF2α) production [25]. The strength of the IFN-τ signal is correlated with the surface size of the embryo(s). Early embryo death occurs when small embryos are unable to produce an adequate signal to prevent the lysis of CL by the luteolytic effect of PGF2α, which is synthesized in the endometrium. The CL produces increased levels of progesterone after ovulation, which has a positive effect on early embryonic development because progesterone stimulates the production of the nutritive substances needed for the growth of the embryo (e.g., polypeptides and mitogenic factors).

According to Leroy et al. [26], the consumption of n-3 PUFAs reduces the formation of PGF2α in the endometrium, which increases the vitality of CL and, thus, the survival of embryos in dairy cows. Chartrand et al. [27] reported a reduction in prostaglandin F2α and E2 (PGF2α, PGE2) in plasma and the uterine fluid when linseed oil (ALA) was supplemented compared to tallow oil during early pregnancy in gilts. The authors concluded that the reduction in prostaglandin synthesis was due to the reduction in ARA and/or the reduction in the ARA substrate in the eicosanoid metabolism.

**Figure 1:**
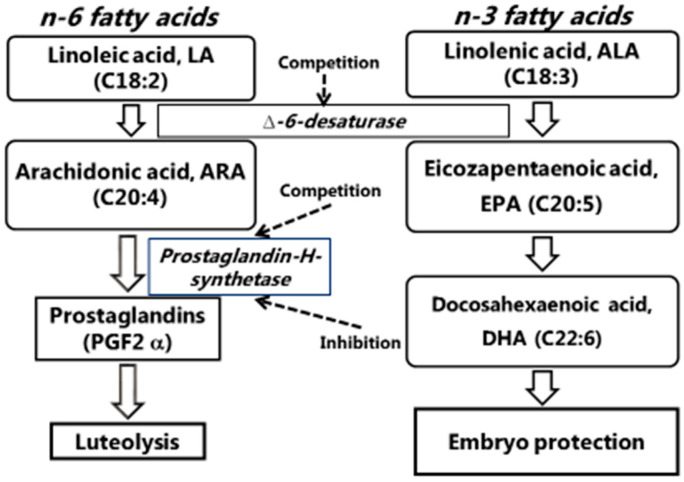
Role of polyunsaturated fatty acids (PUFAs) in reproduction biology.

The precursor of PGF2α is arachidonic acid (ARA, C20:4), which can be derived from the feed but can also be synthesized from LA (C18:2). According to human studies, n-3 fatty acids have an inhibitory effect on the synthesis of PGF2α [28]. ALA competes for the binding sites of Δ6-desaturase responsible for LA to ARA transformation. In contrast, EPA similarly competes for the binding sites of prostaglandin-H-synthase charged for the formation of prostaglandins from ARA. DHA also has a direct inhibitory effect on prostaglandin-H-synthase, as shown in Figure 1.

The feeding of fish oil (EPA and DHA) increases the expression of progesterone receptor mRNA and thus the number of progesterone receptors in the superficial glandular epithelium, which may be beneficial for preparation of the uterus to maintain pregnancy in dairy cows [29].

Although the affinity of Δ6-desaturase for ALA is higher than for LA, the concentrations of LA in animal feed are usually higher, resulting in higher levels of conversion to longer chain n-6 PUFAs. As Δ6-desaturation is a limiting step in the pathway toward the formation of LC PUFAs, a high dietary intake of n-6 PUFAs has been proposed as a limiting factor in the conversion of ALA to EPA and DHA [17]. If the ratio of n-3 fatty acids in the food or feed is increased, this will affect longer chain n-3 fatty acid formation, not only through this increase in absolute levels, but also due to the lower conversion of LA to biologically active metabolites, such as ARA, which is often decreased by increasing the intake of n-3 fatty acids [30].

## 4. LC PUFAs in Practical Swine Nutrition

### 4.1. Effects during the Prenatal Period 

The n-6 fatty acids play many physiologically essential roles in the body. For example, the rate of embryo growth is directly connected to the amount of ARA. Since the presence of EPA and DHA is considered to depress the synthesis of ARA-derived PGs, they may decelerate the growth of embryos at the end of pregnancy and postpone the term of birth. The n-3 LC PUFAs that are unrelated to eicosanoid synthesis can affect myometrial contractions through direct impacts on ion channels and cell signalling, which can also elongate the duration of gestation. This influence of LC PUFAs on the different ion channels of muscle cells is quite well-documented in human models [31]. Several researchers also found that the administration of n-3 LC PUFAs during the gestation period increases the length of pregnancy in swine [32].

According to some studies, n-3 LC PUFAs improved the performance parameters of subsequent parturition when given during lactation or to gilts, thereby suggesting the compensation of a temporary shortage of these nutrients in practical conditions [33,34,35]. The explanation of this could be that follicles start to develop during the actual lactation of sows [36], and the positive effects of n-3 PUFAs on the development and growth of follicles induces larger ova to be ovulated and fertilized after insemination, which can even occur in gilts. These findings are in accordance with the observations of Smit et al. [37] who found larger CL after supplementation with n-3 LC PUFAs in both gilts and weaned sows.

In the study of Rosero et al. [4], ALA supplementation increased the number of sows coming to heat after weaning, reduced the weaning-to-oestrus interval (WOI), enhanced the conception and farrowing rate and decreased the culling rate of sows compared to the unsupplemented controls. In the investigation of Smits et al. [35], 0.33 g/kg n-3 fatty acid treatment reduced the WOI in the experimental group (6.3 vs. 7.8 days); furthermore, the conception and farrowing rate improved by 1% (74.8% vs. 75.8%) and 1.4% (72.1% vs. 73.5%), respectively; hence, the number of live-born piglets (embryo survival) increased by one (9.3 vs. 10.3 live-born). Earlier research showed the positive effects of n-3 LC PUFAs feeding during lactation or in the breeding period compared to the negative control, on the subsequent litter size of sows [33] and first litter size of gilts [34]. However, this was not observed in a more recent study [38]. One possible reason for these inconsistent results could be the improvement of the genetic potential of modern “high-performance” sows. The litter size in the study of Smits et al. [35] was 9.3, while it was 14 in the study of Posser et al. [38]. Another possible reason could be as n-3 LC PUFAs are essential nutrients, the absolute amount of them and the n-6/n-3 LC PUFAs ratios used in the different studies. For example: Rosero et al. [4] used a corn/soybean meal-based diet supplemented with flaxseed oil (ALA); Smits et al. [35] used a wheat/barley-based diet supplemented with fish oil; Webel et al. [33] and Spencer et al. [34] used marine algae as an n-3 source, similar to Posser et al. [38], with the exception that, in the previous two studies, a corn/soybean meal-based diet was used, but the latter was a wheat/barley-based diet. Corn/soybean-based diets contain more n-6 PUFAs than wheat/barley-based diets, so adding n-3 LC PUFAs to a corn/soybean-based diet may increase the n-3/n-6 LC PUFAs ratio to a greater extent, although it does not explain the results of Smits et al. [35]. Rooke et al. [39] observed declining litter size as the amount of salmon oil was incrementally increased in the diet (0, 5, 10, 20 g/kg feed). This finding was unlikely to have been caused by the inclusion of salmon oil from day 60 of gestation, as litter size is determined by ovulation rate and prenatal death, which occurs before day 30 of pregnancy.

Posser et al. [38] found an increased piglet birth weight associated with reduced serum levels in the triglycerides of sows during gestation in the case of 28.0 g/d microalgae (*Schizochytrium* sp.) supplementation, which equated to 3.36 g DHA intake per sow per day. However, several other studies did not demonstrate any effects of n-3 PUFAs supplementation during late gestation on the birth weight of piglets [37,40,41]. However, these observations were made after using PGF2α for the induction of farrowing on days 113–114 of pregnancy, which might have had an opposite effect to that of n-3 PUFAs on the longevity of gestation.

Although Mateo et al. [42] did not find a difference between the birth weights of piglets after feeding on n-3 PUFAs from the second half of gestation and during the lactation period, they did observe a nearly significant difference in the birth weights (1.54 vs. 1.65 kg; *p* < 0.06) for the n-3 PUFA-supplemented group in the subsequent farrowing period.

### 4.2. Effects in the Postpartum Period: Pre-Weaning Growth and Mortality of Piglets

Studies reporting the effects of n-3 PUFAs on the pre-weaning performance of piglets remain controversial [43]. Some researchers found no significant difference between pre-weaning growth or the mortality of piglets when sows were fed with control or n-3 fatty acid-supplemented diets [19,35,44,45]. A very recent study found that litter birth weight and pre-weaning mortality was impaired when 1% salmon oil was added to gestation and lactation diets beside soya oil as a control supplementation [46]. However, some researchers experienced an improvement in the performance parameters of current and subsequent parturition when sows were fed the n-3 PUFAs supplemented diet during the lactation period. Rooke et al. [41] and Mateo et al. [42] reported that piglet weight at weaning was higher than the control if the sows were fed a diet supplemented with n-3 PUFAs from fish oil or marine algae during the second half of gestation. Luo et al. [47] also reported an improved daily weight gain of piglets (0 to 21 days of age) when sows were fed a 70 g/kg fish oil-supplemented diet during lactation. Innis [48] found the same effect in human infants and suggested that n-3 LC PUFA was a pivotal factor for the growth of children. Lavery et al. [49] fed sows with soya and fish oil from day 105 of gestation to weaning and, although there was no effect of oil type on the number of total born, born alive and piglets stillborn, there was a tendency for a reduced pre-weaning mortality rate for sows offered a diet containing salmon oil. These litters also had an increased litter weight gain in the second part of lactation.

Rooke et al. [32] and Farmer et al. [50] found that maternal supplementation with n-3 PUFAs during gestation and lactation reduced pre-weaning mortality. The background of improved mortality of suckling pigs may be that n-3 PUFAs can modulate the immune status of piglets via a significant increase in the n-3 PUFAs content of the immune cells and a reduction in the synthesis of pro-inflammatory eicosanoids [51]. Other possible causes of these effects of n-3 PUFAs on piglet viability and growth could be its beneficial impact on intestinal morphology and its barrier function [52], as there is evidence of better glucose absorption in the jejunum of weanling piglets as an effect of maternal n-3 PUFA supplementation [53]. Additionally, n-3 LC PUFAs can improve not only the gastrointestinal functions and integrity of piglets, but also those of sows. According to Leonard et al. [54], feeding n-3 LC PUFAs to sows from day 109 of gestation until weaning decreased the number of *Escherichia coli* in the cecum and increased villous height in the ileum.

Overall, it can be said that piglets can benefit from the n-3 LC PUFA supplementation of the sow in two ways: first, prenatally, when the developing embryos have access to these fatty acids via placental transport; second, postnatally, when litters consume the colostrum and sow milk containing elevated concentrations of n-3 fatty acids, such as EPA and DHA.

The effects of n-3 LC PUFAs on the performance parameters of sows and their progeny are summarized in Table 1.

## 5. n-3 LC PUFA Sources and Doses in Swine Nutrition

There are different plant and animal sources of n-3 PUFAs, as shown in Table 2, with some differences. For instance, plant oils contain mainly LA and ALA, but n-3 LC PUFAs are present mainly in fish and marine algae oils. Although the n-3 PUFA content of the basic feed ingredients, such as cereal grains, is negligible, rapeseed/canola, soybean, green leafy vegetables, and nuts contain a certain amount of ALA [58]. 

### 5.1. Plant Raw Materials

Echium and linseed/flaxseed oil contain a remarkable amount of ALA [59], but only linseed/flaxseed and theirs oils are used in pig nutrition. In a recent study, flaxseed meal and flaxseed oil supplementation of the lactation diet of sows improved the average daily weight gain (ADGW) and thus weaning weight of piglets to the negative control or fish oil [55].

### 5.2. Animal Raw Materials

Fish oil (salmon, tuna, menhaden oil, etc.) can be used for increasing the n-3 PUFA levels of feeds [60]. Fish oil contains remarkable amounts of both EPA (C20:5n-3) and DHA (C22:6n-3), together accounting for approximately 20% of the total fatty acids [61], although this varies according to fish species, as shown in Table 2. Due to the pressure of declining seafood consumption, the price of fish oil is increasing worldwide, and the demand is partially being replaced by farmed fish oil. This is supported by the fact that the n-3 PUFA content in some farmed fish species is higher than in wild type (e.g., Atlantic salmon (Salmo salar)) [62].

### 5.3. n-3 LC Supplements

Marine algae oils are also used to increase the n-3 PUFA levels of feeds [54] but, due to their high price, only as feed supplements in low amounts. Marine algae, such as Schizochytrium, Thraustochytrids, Aurantiochytrids, Oblongiochytrids, and Aplanochytrids species, mainly contain DHA but are also a moderate source of ALA and EPA [38,63].

### 5.4. Dosing of n-3 LC PUFA

Many researchers have investigated the appropriate dose of n-3 PUFAs for swine feed. It seems that, besides the proportion of n-6 and n-3 PUFAs, the absolute amount of different fatty acids is important. In the global swine industry, a high ratio between n-6 and n-3 PUFAs is common, due to the feed ingredients used, such as corn, while it can be much lower in other ingredients, such as soybean or rapeseed.

The experimental results for ALA supplementation in feeds for sows gives a puzzling picture. Still, it appears that higher doses give better results, and the source of n-6 PUFAs is also essential in the diet. Gunnarsson et al. [56] fed sows with linseed oil from weaning to weaning at a dose of 10 g/kg feed, and thus the n-6/n-3 ratio of the control (11.04) was reduced to 1.95 in the n-3 PUFA group. They found no significant effect of the treatment on litter size nor piglet birth weight. De Quelen et al. [19] used linseed oil at a 15 g/kg dose in gestation and a 55 g/kg dose in lactation groups without noting any positive effects regarding litter size, mean birth weight of live piglets or ADWG of piglets. Farmer et al. [50] found a positive effect of feeding 10 g/kg flaxseed on pre-weaning mortality compared to the negative control. Rosero et al. [4] investigated the effect of a different combination of LA and ALA (n-6/n-3 ratios varied 22:1 to 5:1) on the performance of subsequent parturition (LA: 21, 27, 33 g/kg feed; ALA: 15, 30, 45 g/kg feed) and experienced the best results with the highest doses of both LA and ALA. On the contrary, Eastwood et al. [55] found that flaxseed meal and the oil supplementation of the lactation diet was most beneficial concerning the ADWG and weaning weight of piglets when the n-6/n-3 ratio was 7 or 5, opposite to 1 or the negative control (n-6/n-3 ratio: 7) or salmon oil (n-6/n-3 ratio: 5) supplementation.

Rooke et al. [32,41] used fish oil (from salmon or tuna) in their experiments using doses of 16.5–17.5 g/kg in the feed. They found some positive effects on the pre-weaning mortality or the growth rate of suckling pigs. Papadpoulos et al. [45] used 20 g/kg feed of fish oil compared to a mixture of sunflower and soybean oils during the lactation period. They observed no effect on piglet performance, even though the n-6/n-3 ratio reduced from 10.13 to 2.09. Matteo et al. [42] fed 2 g/kg of protected (encapsulated) fish oil in the second phase of gestation and during the lactation of gilts with positive results on the birth weight and ADWG of piglets, which may indicate that a lower amount of n-3 LC PUFA could result in positive effects when the source and time of feeding are appropriately chosen. Leonard et al. [54] supplemented sows’ diets from day 109 of gestation to weaning with a combination of seaweed oil (10 g animal/day) and fish oil (100 g/animal/day), resulting in a high dose of n-3 LC PUFAs (approximately 40 g EPA and 25 g DHA/animal/day). They did not find an effect on the performance parameters of animals. However, dietary fish oil supplementation enhanced the n-3 PUFA proportion of sow milk and piglet serum at weaning. Smits et al. [35] used salmon oil at a dose of 3.3 g/kg of feed during the lactation period, observing improved parameters during the subsequent farrowing. In this trial, the n-6/n-3 ratio was reduced from 15.1 to 9.5, indicating that a n-6/n-3 ratio of about 10:1 is already sufficient for producing positive results. In a later study, Smits et al. [57] used different doses of fish oil (3 and 10 g/kg feed) in the diets of gilts and found that lower doses of fish oil gave better results on embryo survival than higher doses. These results suggest that, in some instances, a lower dose of n-3 PUFA and an intermediate n-6/n-3 ratio would be preferred.

There are several studies where n-3 LC PUFA from marine algae were used as the supplement. Webel et al. [33] and Spencer et al. [34] used 85 g of marine algae extract (MAE) and observed favourable results on embryo survival and subsequent litter size. This MAE contained 120 g/kg of DHA, which equated to more than 10 g of DHA per animal per day in those experiments, which is equivalent to approximately 100 g fish oil. Smit et al. [37] used almost the same dose (84 g/animal/day) of the same MAE product, which was fed in the second half of gestation until weaning, and did not report any positive effects on the performance parameters of primiparous sows and their piglets, but observed an enhancement in the CL size of the supplemented group. In a later study, Smit et al. [40] did not find any difference between the control and supplemented groups of sows fed from weaning to weaning, although they used a much lower dose of MAE (3 g/kg feed). Posser et al. [38] fed a different amount of the same MAE source in the late gestation period until the next artificial insemination (AI). They found a positive impact on the subsequent litter weight at the highest tested dose (28 g/animal/day) during this experiment, which was equivalent to 3.36 g DHA per day. According to the studies mentioned above, MAE seems to have a balanced performance on swine reproduction, which appears to be positively dose-dependent.

## 6. Conclusions

According to human, animal and field studies, it seems that n-3 PUFAs, fed during the gestation period, lead to increased lengths in pregnancy times due to a negative impact on ARA production, which exhibits a linear correlation with embryo size. Without the induction of farrowing (e.g., with PGF2α), n-3 PUFA administration may not negatively influence the birth weights of piglets, as foetuses have more time (some days) to develop before parturition. Supplementation with n-3 PUFAs during the lactation period may improve the quality of ovarian follicles, thereby improving the survival rate of embryos and the subsequent litter size, as was demonstrated by several studies, when there was an ongoing subclinical deficiency in this nutrient. These biological reproductive effects will be reinforced if supplementation is elongated until the end of the first trimester of gestation when ovulation, fertilization, and implantation occur, and the skeletal frame of embryos develop. During this period, the effect of n-3 PUFA on follicle maturation and progesterone synthesis via CL size could help to maintain the pregnancy and reduce the rate of early embryo loss, which is specific to pigs.

Besides reproduction aspects, n-3 LC PUFAs are also transferred into the bodies of suckling piglets through colostrum and sow milk, helping to provide these essential nutrients of piglets in high-performance modern herds.

In case of deficiency, it seems that n-3 PUFAs could have favourable impacts on performance parameters during the whole production cycle of the sow, but especially during the lactation period, when piglets could also benefit from the supplementation directly. In other production periods, the dose, basal sow diet, and n-6/n-3 PUFA ratio may have a significant influence on the outcome.

As there are no current feeding recommendations available for PUFAs in swine nutrition, more research is required with standardized metabolic trials and comparing the different practical feeding situations for the establishment of PUFAs, including n-6/n-3 ratio requirements. More studies should be performed to find a threshold for PUFAs requirements that does not improve production and reproduction results further. The appropriate dose of n-3 PUFA in sow nutrition and the n-6 PUFA levels in the feeds must be taken into account to ensure that there are no significant reductions in the n-6/n-3 ratio. Despite the previous field trials, further examinations will be needed to certify the assumptions and conclusions of this review.

## Figures and Tables

**Table 1:** Effects of n-3 long-chain PUFAs on performance parameters of sows and their piglets.

Reference	Oil Source	Time of Feeding	Litter Size	Litter Weight	Piglet Weight Gain	Piglet Mortality	Sow Performance
Rosero et al. [4]	LO (different LA and ALA ratios)	Lactation	No effect	No effect	Increased	No effect	Improved WOI, heating %, and farrow %
De Quelen et al. [19]	LO 15 and 55 g/kg feed	Gestation and lactation	No effect	No effect	No effect	No effect	No effect
Rooke et al. [32]	FO (salmon) 1.65 g/kg feed	Gestation and lactation	No effect	Reduced individual weight	Increased	Decreased	
Webel et al. [33]	MA 85 g/sow/d	Five days prior parturition to breeding	Increased subs. litter size	-	-	-	No effect
Spencer et al. [34]	MA 85 g/sow/d	30 days prior breeding to farrow	Increased litter size	Reduced individual weight	-	-	-
Smits et al. [35]	FO 0.33 g/kg feed	Lactation	Increased subs. litter size	-	No effect	No effect	Improved WOI and farrow %
Smit et al. [37]	MA 84 g/sow/d	60th day of gestation to weaning	No effect	No effect	No effect	Increased	No effect, larger CL
Posser et al. [38]	MA 3.5, 7, 14, 28 g/sow/d	85th day of gestation to breeding	No effect	Improved at the highest dose	No effect	-	No effect
Rooke et al. [39]	FO (Salmon) 5, 10, 20 g/kg feed	60th day of gestation to farrow	Reduced as FO dose increased	No effect	-	No effect	-
Smit et al. [40]	MA 5 g/kg feed	Weaning to weaning	Reduced	No effect	No effect	-	-
Rooke et al. [41]	FO (Tuna)17.5 g/kg feed	63rd to 91st day of pregnancy	No effect	No effect	Increased	No effect	-
Matteo et al. [42]	FO 2 g/kg feed	60 days before parturition to weaning	Increased subs. litter size	Increased in subs. farrow	Increased	No effect	No effect
Lauridsen and Jensen [44]	FO 80 g/kg feed	One week prior to farrow and lactation	-	-	No effect	-	-
Papadopoulos et al. [45]	FO 20 g/kg feed	Eight days before farrow to weaning	-	-	No effect	No effect	-
McDermott et al. [46]	FO (Salmon) 1% of feed	Gestation and lactation	No effect	Decreased	No effect	Increased	First par. impaired Second par. improved
Luo et al. [47]	FO 70 g/kg feed	Lactation	-	No effect	Increased	-	-
Lavery et al. [49]	FO 1.8 and 6%	105th day of gestation to weaning	-	No effect	Increased	Decreased	-
Farmer et al. [50]	FSM and FSO 100 g/kg feed	63rd day of gestation to weaning	-	No effect	No effect	Decreased	No effect
Leonard et al. [54]	SWE 10 g/sow/d + FO 100 g/sow/d	109th day of gestation to weaning	-	-	No effect	-	-
Eastwood et al. [55]	FSM and FSO, FO	80th day of gestation to weaning	-	No effect	Increased	No effect	-
Gunnarsson et al. [56]	LO 10 g/kg feed	Weaning to weaning	No effect	No effect	-	-	-
Smits et al. [57]	FO 3 and 10 g/kg feed	6 weeks before and 25 days after breeding	No effect	-	-	-	Increased embryo survival

FO = Fish oil; MA = Marine algae; LO = Linseed oil; WOI = Weaning-to-oestrus interval; CL = Corpus luteum; LA = linoleic acid; ALA = alpha-linolenic acid; FSM = Flaxseed meal; FSO = Flaxseed oil; SWE = Seaweed extract.

**Table 2:** Different sources of long-chain polyunsaturated fatty acids (LC PUFAs) in animal husbandry (adapted from [17]).

Oil Source	LA (18:2n-6)	ALA (18:3n-3)	EPA (20:5n-3)	DHA (22:6n-3)
g/kg DM				
Rapeseed oil	180	90	-	-
Echium oil	150	310	-	-
Soybean oil	530	80	-	-
Linseed oil	160	530	-	-
Menhaden oil	11	15	122	79
Herring oil	11	7	68	58
Tuna oil	15	4	57	224
Salmon oil	12	6	120	138
Marine algae	-	27	8	250

LA = linoleic acid; ALA = alpha-linolenic acid; EPA = eicosapentaenoic acid; DHA = docosahexaenoic acid; g/kg DM = gramm per kilogramm dry matter.
